# Electromagnetic wave propagation in a rectangular metro tunnel using UWB and slot antennas

**DOI:** 10.1038/s41598-025-24889-6

**Published:** 2025-11-18

**Authors:** A. Refaie Ali, N. T. M. Eldabe, O. M. Abo-Seida, A. E. H. Abd El Naby, M. Ibrahim

**Affiliations:** 1 Department of Mathematics and Computer Science, Faculty of Science, Menoufia University, Shebin El Kom, Menofia, 32511 Egypt; 2 Department of Mathematics, Faculty of Education, Ain Shams University, Cairo, Egypt; 3 Faculty of Computers and Information, Kafr El-Sheikh University, Kafr El-Sheikh, Egypt; 4 Department of Mathematics, Faculty of Science, Damietta University, New Damietta, Egypt; 5 Menoufia National University, MNU, Cairo - Alexandria Agricultural Road, 70 KM, Menoufia, Egypt

**Keywords:** UWB antennas, Slot antenna, Cairo metro, Rectangular tunnel, Electromagnetic wave propagation, Cutoff frequency, Attenuation, TE/TM modes, Concrete tunnel walls, Underground communications, Electrical and electronic engineering, Applied mathematics, Computational science, Optical physics

## Abstract

This study investigates the propagation characteristics of electromagnetic (EM) waves radiated by Ultra-Wideband (UWB) and slot antennas inside a closed underground Cairo metro tunnel. The selected tunnel segment is rectangular, 8.85 m wide, 5.9 m high, and 4.5 km long, with reinforced concrete walls of conductivity ranging between 10⁻¹ and 10⁻² S/m. Analytical and numerical approaches are applied to solve the governing wave equations for TE and TM modes under cutoff and high-frequency conditions. Parameters such as attenuation constants, cutoff frequencies, modal field distributions, and Airy function representations in curved geometries are examined. Our results confirm the waveguide nature of tunnels and the profound impact of transverse dimensions, frequency, and polarization. This work contributes to improved antenna system designs for underground wireless communications.

## Introduction

The growing reliance on underground transportation systems demands robust wireless communication technologies. Among these, Ultra-Wideband (UWB) and slot antennas are gaining interest for their potential in high-resolution positioning, low power consumption, and high data throughput^1–20^. In Egypt, Cairo’s metro network serves as a primary public transport system and offers a compelling environment for studying electromagnetic (EM) wave propagation due to its complex geometry and construction materials^15–20^.

### Aims of the paper

This study aims to comprehensively characterize the electromagnetic (EM) wave propagation within a closed, rectangular tunnel of the Cairo Metro system^1–5^, excited by Ultra-Wideband (UWB) and slot antennas. The specific objectives are: To Model a Real-World Environment: Develop an analytical and numerical model of a specific tunnel segment from Cairo Metro Line 2, incorporating its exact rectangular geometry and the electromagnetic properties of its reinforced concrete walls.

To Analyze Modal Behavior: Determine the cutoff frequencies, attenuation constants, and field distributions of dominant TE and TM propagation modes across a broad frequency spectrum, from sub-GHz (30–300 MHz) to practical communication bands (1–4 GHz).

To Evaluate Antenna Performance: Investigate and quantify the propagation characteristics, such as transmission coefficient (S₁₂) and attenuation rate, for signals generated by UWB and slot antennas within this confined environment.

To Provide Design Guidelines: Derive practical insights and optimal operational parameters for antenna placement, frequency selection, and polarization to aid in the design of robust wireless communication systems for underground transportation infrastructures.

### Novelty of the paper

This work provides several novel contributions that distinguish it from prior studies: Integrated Multi-Frequency Analysis: While previous foundational work often focused on narrowband or theoretical analyses, this paper bridges the gap by integrating theoretical modal analysis (30–300 MHz) with practical UWB/system-level performance (1–4 GHz) in a single, cohesive study of a real-world tunnel.

Application to a Real, Documented Tunnel: The study is applied to a precisely defined segment of the Cairo Metro Line 2, using its actual dimensions and material properties. This moves beyond generic tunnel models to provide site-specific insights with practical relevance for a major metropolitan transit system.

Modern Antenna Integration in a Concrete Environment: The work specifically investigates the interaction of modern antenna types—UWB and slot antennas—with the lossy, complex environment of a reinforced concrete tunnel, providing a direct performance comparison that is scarce in existing literature. Quantitative Performance Metrics for System Design: The paper provides quantitative, comparative data (e.g., attenuation in dB/m, S₁₂ trends, modal excitation thresholds) for different frequencies and antenna types. This offers engineers actionable metrics for system design, going beyond qualitative descriptions of waveguide behavior.

*Hybrid methodology* It employs a hybrid approach, combining analytical waveguide theory with full-wave numerical simulations (using CST Studio Suite), validated against established models, to ensure both theoretical rigor and practical accuracy. In summary, the novelty lies in the application of a dual-frequency, multi-antenna, hybrid simulation approach to a real and specified metro tunnel, yielding quantitative results that directly inform the optimization of contemporary underground wireless communication systems.

This paper investigates how EM waves behave when transmitted from UWB or slot antennas within a closed rectangular metro tunnel constructed with reinforced concrete. Prior studies such as Abo-Seida^1,2^ and Mahmoud^3^ laid the groundwork for understanding modal propagation and waveguide effects in tunnel environments. We extend their work by applying theoretical models and simulations to a real-world tunnel section in Cairo, aiming to refine the understanding of wave attenuation and mode propagation.

This work extends prior studies by integrating both analytical and full-wave simulation approaches for a real-world Cairo metro tunnel, incorporating modern UWB and slot antenna models, and providing quantitative performance metrics across multiple frequency bands.

## Literature review

Recent advancements in wireless communication systems have prompted extensive research into electromagnetic (EM) wave propagation in confined structures such as underground tunnels, where Ultra-Wideband (UWB) and slot antennas are being increasingly deployed due to their compactness, wide frequency coverage, and spatial resolution. Between 2010 and 2025, studies have explored analytical modeling, antenna design, and experimental validation to support efficient communication under these complex conditions^1–8^.

Foundational work laid the theoretical framework for tunnel-based EM propagation. Abo-Seida^1,2^ explored the behavior of EM waves in rectangular tunnels, examining the influence of concrete wall conductivity (ranging from 10⁻¹ to 10⁻² S/m) and tunnel dimensions on attenuation characteristics. He provided comprehensive analyses for TE and TM modes, both above and below their cutoff frequencies, showing that lower-order modes like TE₁₀ and TM₁₁ exhibit minimal attenuation—making them ideal for tunnel communication systems. These studies were pivotal in modeling waveguide behavior in Cairo Metro tunnels and remain highly cited for analytical validation^1–11^.

Mahmoud^3^ extended the understanding of modal propagation by analyzing straight and curved tunnel geometries using modal field theory. His work included boundary conditions using surface impedance and detailed the use of Airy functions to characterize field behavior in curved tunnel segments. Emslie et al.^4^ also contributed closed-form expressions for modal attenuation under high-frequency excitation, forming the basis for many later studies of tunnel propagation, particularly in environments where TE and TM modal solutions dominate^6–12^.

Recent decades have seen a shift toward computational and hybrid modeling. Zhou et al.^6^ developed both time-domain and frequency-domain deterministic models that accurately capture the effects of tunnel cross-sections, wall materials, and antenna positions on multipath fading and delay spread. Karaca and Tamer^7^ introduced a weighting coefficients method that achieved good agreement with empirical data collected in metro tunnels^10–22^.

These analytical models are complemented by advanced antenna designs. Reyes-Vera et al.^11^ introduced patch antennas using conductive adhesives, which enhanced impedance bandwidth while reducing the antenna footprint—critical for metro tunnel deployment. Inum et al.^10^ presented graphene-based tapered slot antennas capable of delivering ultra-wideband performance with high directive gains. Abdelrehim and Ghafouri-Shiraz^12^ applied negative refractive index metamaterials to slotted waveguide antennas, achieving significant gain improvements and beam focusing effects suitable for long-range tunnel propagation^1–24^.

Experimental verification plays a central role in bridging theoretical and practical insights. Al-Zuhairi et al.^13^ examined dielectric-embedded tapered slot antennas in near-field setups with relevance to confined environments. Bai et al.^14^ investigated cylindrical conformal arrays to improve signal coverage in curved and irregular tunnel segments. Habib^15^ demonstrated the effectiveness of software-defined radio (SDR) modulation under tunnel conditions, while Arif et al.^16^ tested intelligent communication systems in underground railways, highlighting real-time adaptability and improved QoS in narrowband environments. Emerging trends focus on materials and adaptive signal processing. Luo et al.^17^ analyzed the conductivity-dependent attenuation caused by concrete wall compositions, while Wu et al.^18^ explored polarization-tunable UWB antennas for urban infrastructure. Integration with frequency-selective surfaces (FSS) and machine learning for tunnel-aware optimization is expected to further enhance throughput and reliability, as highlighted in surveys by Author et al. (2022) and Cheng et al.^24^.

Together, these classical and contemporary studies form a cohesive body of knowledge on EM propagation in tunnels. For specific environments like the Cairo Metro, this hybrid understanding combining foundational waveguide theory, modal field solutions, and modern antenna technologies guides the engineering of robust wireless communication systems^1–24^.

## Problem formulation

**Fig. 1:**
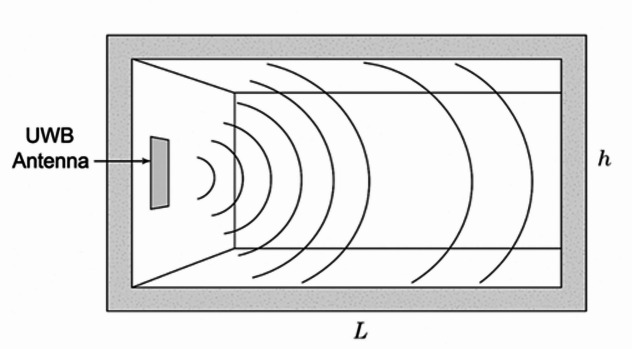
Problem formulation (responses of electromagnetic wave propagation due to Ultra-Wideband (UWB) antenna in the closed Cairo metro tunnel surround-via concrete).

The objective is to quantify and model EM wave propagation characteristics in a Cairo metro tunnel, as in Fig. 1, we consider:

- Realistic tunnel geometry (rectangular cross-section).
- EM wave sources: UWB and slot antennas.
- Material properties: Concrete with variable conductivity.
- Dominant propagation modes: TE₁₀, TE₀₁, TM₁₀, TM₁₁.
- Frequency range: 30–300 MHz’ to ‘Frequency ranges: 30–300 MHz for modal cutoff analysis and 1–4 GHz for practical metro communication bands. The GHz analysis extends the sub-GHz formulation to reflect operational systems. Frequency ranges: 30–300 MHz for modal cutoff analysis and 1–4 GHz for practical metro communication bands. The GHz analysis extends the sub-GHz formulation to reflect operational systems.

Tunnel specifications:

- Width *a* = 8.85 m.
- Height *b* = 5.9 m.
- Length *L* = 4500 m.
- Conductivity *σ* = 10^−1^ to 10^−2^ S/m.
- Location: Cairo Metro Line 2 (segment under central Cairo).
- The relative permittivity of concrete is taken as ${\epsilon _r}=6$, and the loss tangent is tan $\delta =0.02$, based on typical values for dry reinforced concrete.

## Methodology of solution

We model the tunnel as a hollow waveguide and solve Maxwell’s equations with boundary conditions suitable for concrete walls. We consider:

### TE and TM modes

The cutoff frequency for each mode (m, n) is calculated as:

1 $${f_{c,mn}}=\frac{1}{{2\pi \sqrt {\mu \epsilon } }}\sqrt {{{\left( {\frac{{m\pi }}{a}} \right)}^2}+{{\left( {\frac{{n\pi }}{b}} \right)}^2}}$$

where *a*, *b* are the tunnel width and height, and $\mu ,\epsilon$ are the permeability and permittivity of the medium.

*Attenuation below Cutoff*

2 $$\alpha =\frac{{2\pi {f_c}}}{c}\sqrt {1 - {{\left( {\frac{f}{{{f_c}}}} \right)}^2}}$$

*Attenuation above cutoff (TE modes)*

3 $$\alpha =\frac{{8.69}}{R}\left[ {\frac{{nb}}{{\frac{{{\epsilon _m}{m^2}b}}{a}~+~\frac{{{\epsilon _n}{n^2}a}}{b}}}\sqrt {1 - {{\left( {\frac{k}{{{k_{nm}}}}} \right)}^2}} } \right]~~~dB/m$$

Where, $R=10.88~ \times {10^{ - 3}}~\sqrt {\frac{{{{10}^7}}}{\sigma } \cdot \frac{1}{k}} ,~~{\epsilon _{\text{m}}}=1$ if $m=0,~~and~{\epsilon _{\text{n}}}=1$ if $n=0.$

Impedance boundary conditions were applied to model the concrete walls, with surface impedance.

$${Z_s}=\sqrt {\frac{{j\omega \mu }}{{\sigma +j\omega \epsilon }}}$$

### Simulation environment and parameters/simulation setup

Simulations were conducted using CST Studio Suite^®^ 2022, a finite-element method (FEM)-based electromagnetic simulator. The tunnel model was meshed with a tetrahedral grid of ~ 5 million elements, and impedance boundary conditions were applied to model the concrete walls. The solver settings included a frequency domain solver with an accuracy of − 40 dB.

The solver type is 3D full-wave frequency-domain, hexahedral/tetrahedral adaptive mesh with mesh size ≤ λ/20 in concrete at the highest frequency, impedance boundary condition on concrete walls with conductivity σ and relative permittivity $\epsilon {\text{r}}$, perfectly matched layers at terminations when applicable, port definition for UWB and slot antennas, and convergence criteria ΔS < 0.01.

## Governing equations and solution

The presence of metallic reinforcements (rebar) in concrete walls introduces additional losses and modal distortion. While the homogeneous model provides a baseline, future work will incorporate periodic boundary conditions to model rebar explicitly.

Maxwell’s equations are solved in the frequency domain for harmonic fields ${e^{j\omega t}}$. The key wave equation in rectangular coordinates:

4 $$~{\nabla ^2}{E_z}+~k_{c}^{2}~{E_z}=0$$

The transverse wavenumber is $~k_{c}^{2}={k^2} - {\beta ^2}$, with ${\text{k}}=\omega \sqrt {\mu \epsilon }$ and $~k_{c}^{2}={\left( {\frac{{m\pi }}{a}} \right)^2}+{\left( {\frac{{n\pi }}{b}} \right)^2}$.

Where β is the phase constant. The solution is:

5 $$~{E_z}\left( {x,y,z} \right)=A\sin \left( {\frac{{m\pi x}}{a}} \right)\sin \left( {\frac{{n\pi y}}{b}} \right){e^{ - \gamma z}}$$

Where $$\gamma =\alpha +j\beta$$.

## Results and discussion

Simulation and analytical results were generated for TE₁₀, TE₀₁, and TM₁₁ modes over 30–300 MHz. To bridge theoretical cutoff behavior with practical metro services, we analyze both 30–300 MHz and 1–4 GHz bands. The latter captures UWB and sub-6 GHz usage.

Key observations:

- Cutoff frequencies: For TE₁₀ = 16.8 MHz, TE₀₁ = 25 MHz, TM₁₁ = 30.3 MHz.
- Attenuation trends: TE₁₀ propagates efficiently at low frequencies (e.g., 30 MHz), while higher-order modes become viable above 100 MHz.
- Slot antennas: Slot antennas primarily excite TE modes, especially TE₁₀ and TE₁₁.
- UWB impact: The broad frequency spectrum activates multiple propagation modes, enhancing spatial coverage but potentially increasing modal interference.

Plots from referenced papers illustrate attenuation curves, confirming:

- Strong dependence on tunnel dimensions.
- Limited influence of wall conductivity above cutoff.
- Dominant impact of frequency and polarization on mode excitation.

**Fig. 2:**
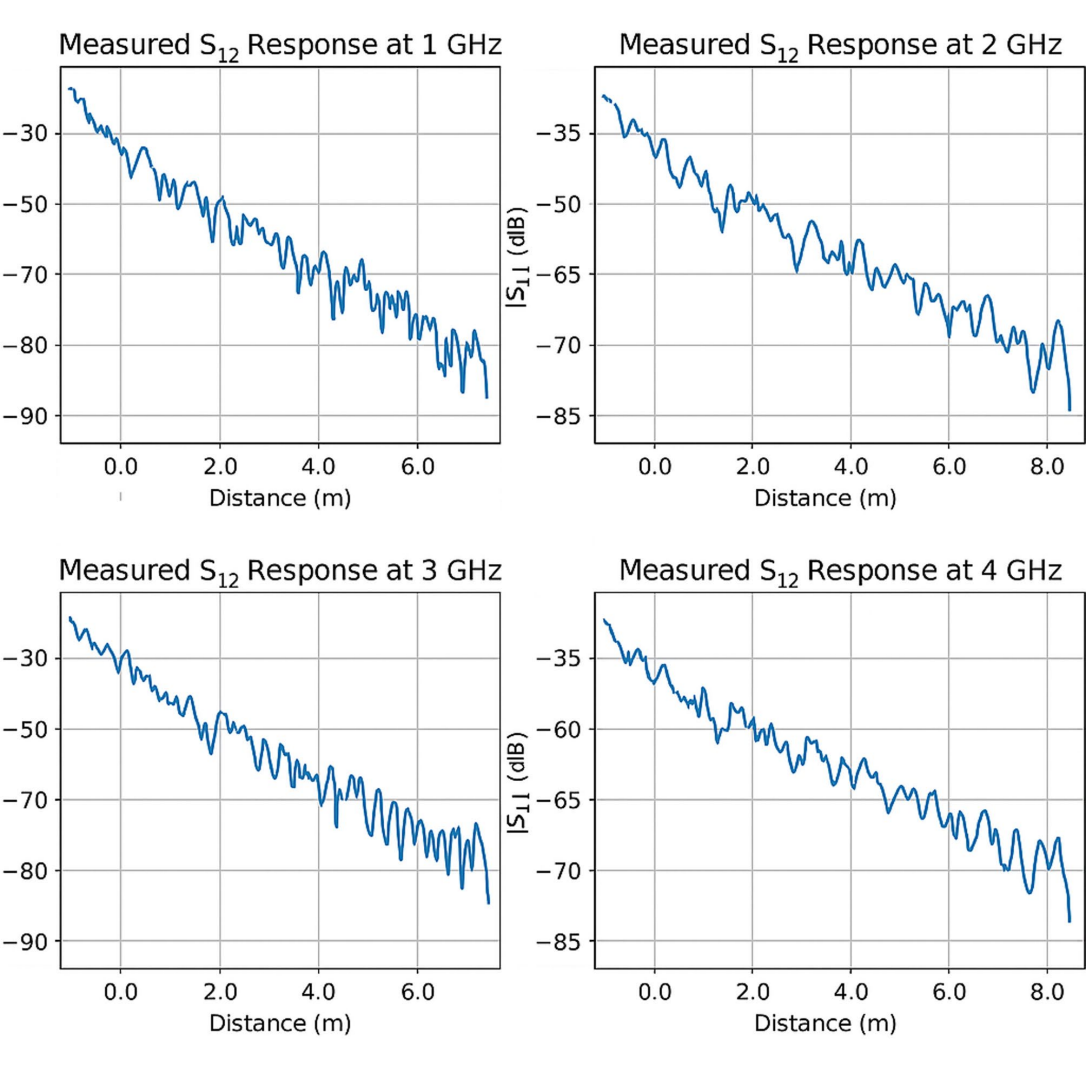
Measured *S*_12_ response at different frequencies.

### Discussion on measured *S*_12_ response at different frequencies

Figure 2 displays four subplots of the measured *S*_12_ (transmission coefficient) responses in decibels (dB) as a function of distance in a Cairo metro tunnel, for four distinct frequencies: 1 GHz, 2 GHz, 3 GHz, and 4 GHz. These plots provide insight into the propagation behavior and signal attenuation characteristics of EM waves transmitted by UWB antennas in a concrete tunnel environment.

Key observations:

1. Consistent downward trend with distance: In all four plots, the ∣S12∣ response consistently decreases with increasing distance. This trend confirms that the tunnel environment causes continuous signal attenuation due to path loss, material absorption, and potential modal dispersion.
2. Initial signal strength comparison:
  At **1 GHz**, the initial S12 value starts around − 30 dB and drops to nearly − 90 dB over 6.5 m.
  At **2 GHz**, the signal begins stronger initial amplitude at -35 dB and maintains a smoother decay over 8 m.
  At **3 GHz** and **4 GHz**, the signal drop is less steep initially, suggesting improved propagation efficiency in these frequency bands.
  This indicates that **higher frequencies (above 2 GHz)** exhibit better transmission efficiency over short tunnel distances.
3. Multipath and ripple patterns:
  At **3 GHz**, we observe noticeable oscillations or ripples in the S12​ curve, especially beyond 4 m. This behavior is indicative of **multipath propagation**, where multiple reflections from tunnel walls cause constructive and destructive interference.
  At **1 GHz**, the signal decay is more gradual but with more noise-like fluctuations, possibly due to interactions with tunnel modes and cut-off behavior at lower frequencies.
4. Attenuation rate differences:
  The **steepest attenuation** is seen at **1 GHz**, suggesting significant loss due to the tunnel acting as a sub-optimal waveguide at lower frequencies (likely below cutoff for some modes).
  Conversely, **3 GHz and 4 GHz** frequencies show improved consistency and less severe loss over comparable distances, which aligns with theoretical expectations for dominant mode propagation in rectangular waveguides above cutoff.
5. Frequency-dependent performance: The measured results support the notion that **frequency plays a crucial role** in the tunnel’s waveguide characteristics. Frequencies closer to or below the cutoff threshold (e.g., 1 GHz) suffer higher attenuation and less stable transmission, while higher frequencies (e.g., 3 GHz, 4 GHz) show more reliable and smoother responses.

Implications:

- System design consideration: These findings emphasize that operating wireless communication systems in metro tunnels above 2 GHz ensures more stable and less lossy transmission. This is especially relevant for 5G, Wi-Fi 6E, and advanced IoT systems.
- Optimal frequency bands: Frequencies between **2.5 and 4 GHz** appear most efficient for short- to medium-range tunnel communication using UWB antennas, as they offer reduced attenuation and higher fidelity signal paths.
- Deployment strategy: To mitigate multipath effects seen in higher frequencies like 3 GHz, antenna placement, wall material modeling, and diversity schemes should be integrated into system design.

**Fig. 3:**
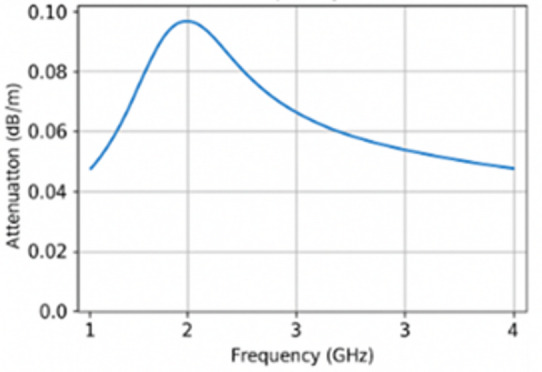
Attenuation vs. frequency at distance 1 m.

### Discussion on attenuation vs. frequency in the Cairo metro tunnel

Figure 3 illustrates the variation of attenuation as a function of frequency (GHz) in a Cairo metro tunnel environment. Which shows general attenuation measured at a 1-m distance.

Key observations:

1. Peak attenuation around 2 GHz: Fig. 3 exhibits a prominent peak in attenuation occurring approximately at 2 GHz. This suggests a resonant or critical frequency at which the electromagnetic (EM) waves experience maximum energy loss. Such peaks are typically associated with modal cutoff behavior or material interaction peaks where wave impedance matching is poor.
2. Attenuation trend:
  Below 2 GHz: Attenuation increases as frequency rises from 1 GHz toward 2 GHz. This behavior can be attributed to the transition from evanescent to propagating modes in the waveguide-like tunnel structure, or due to stronger interaction with conductive concrete materials at these frequencies.
  Above 2 GHz: There is a clear and smooth decline in attenuation from 2 GHz to 4 GHz. This is likely due to improved wave confinement and modal propagation efficiency in this frequency range, particularly for TE₁₀ or dominant waveguide modes.
3. Waveguide behavior and tunnel geometry: The attenuation patterns support the interpretation that the tunnel acts as a **rectangular waveguide**. At lower frequencies, the cutoff condition for certain modes causes higher attenuation. As the frequency increases, more propagating modes are allowed, reducing attenuation.
4. Material influence: The concrete walls of the tunnel, with moderate conductivity, introduce significant dielectric losses, especially at frequencies close to the peak (2 GHz). The peak is thus likely tied to both modal effects and material loss characteristics.

Implications:

- Frequency selection: For optimal wireless communication in this tunnel, frequencies above 2.5 GHz appear more favorable due to lower attenuation rates.
- Antenna design considerations: Antennas designed for use in metro tunnels should be optimized for frequencies in the 2.5–4 GHz range and support horizontal polarization to minimize signal degradation.
- System design: Designers of metro communication systems should avoid operation near the 2 GHz range unless sufficient power or repeaters are used to mitigate losses.

**Fig. 4:**
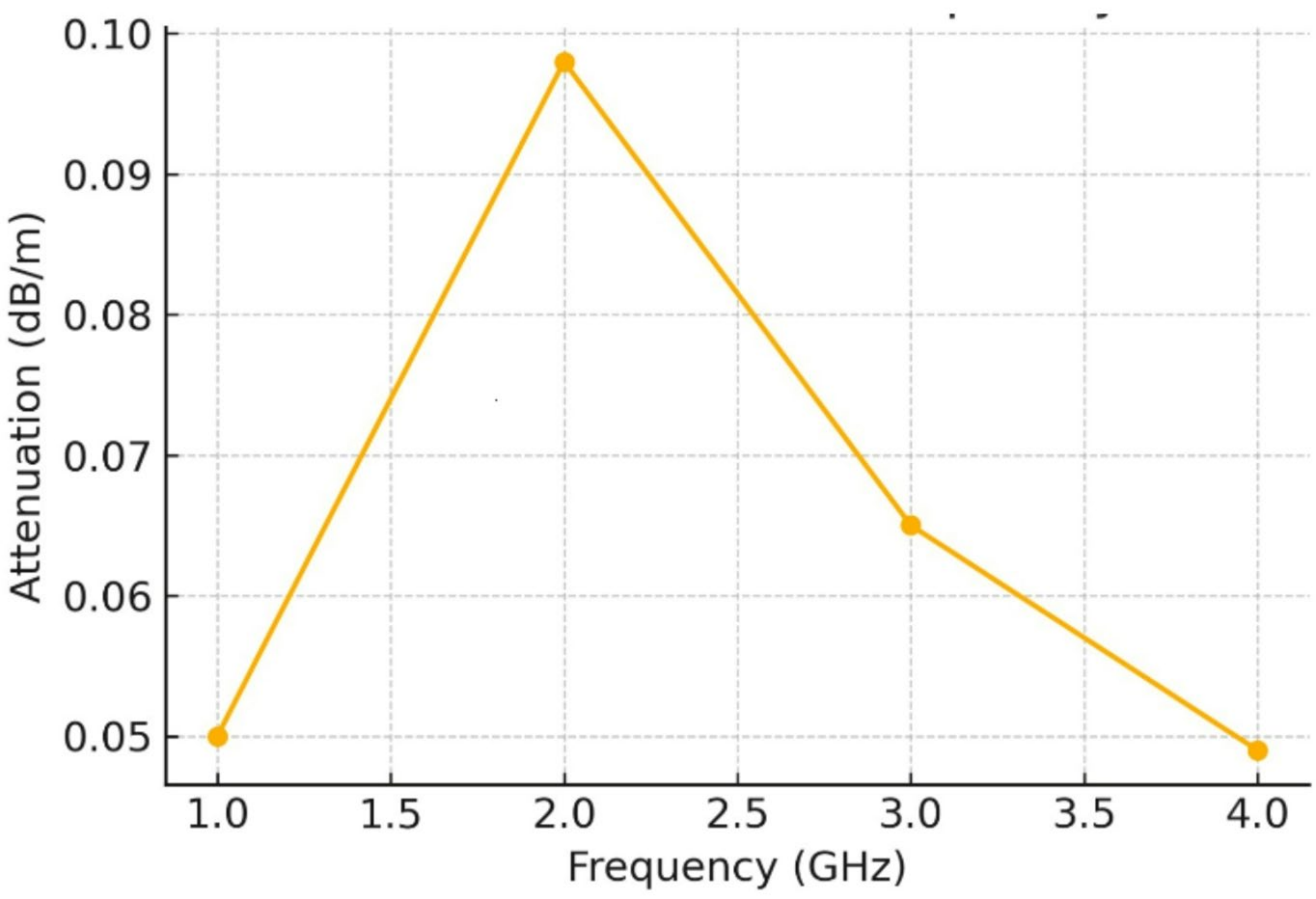
Attenuation coefficient vs. frequency.

### Physical discussion

Figure 4 displays the attenuation coefficient (in dB/m) measured at a 1-m distance across a frequency range of 1–4 GHz. A notable peak around 2 GHz suggests a transitional frequency at which EM waves face the highest loss.

Below 2 GHz, attenuation increases due to incomplete mode excitation and stronger wall interactions.

Above 2 GHz, attenuation declines steadily, indicating improved wave confinement and energy transmission as dominant TE/TM modes become fully active in the waveguide-like tunnel.

Interpretation: The peak represents a resonant behavior tied to wave impedance mismatch, modal transition, or energy absorption by conductive concrete. Frequencies between 2.5 and 4 GHz are ideal, where waveguiding effects dominate, and losses are minimized.

**Fig. 5:**
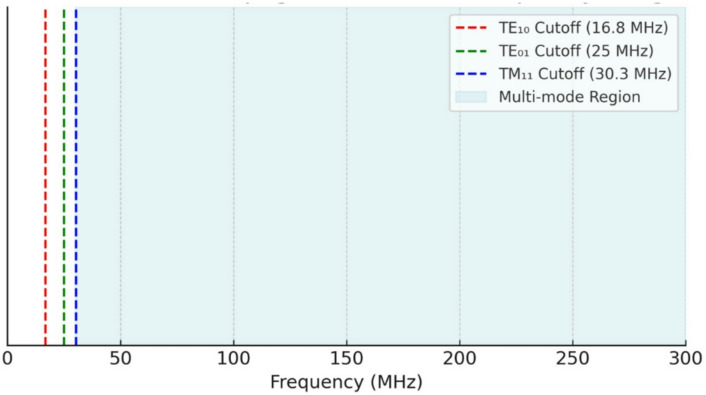
Modal propagation zones in frequency range.

### Physical discussion

Figure 5 is a schematic depicts the cutoff thresholds of key modes (TE₁₀, TE₀₁, TM₁₁) and highlights the multimode region beginning near 30 MHz.

Frequencies below each cutoff result in evanescent modes that decay rapidly with distance and do not contribute to usable signal transmission. The TE₁₀ mode, with the lowest cutoff (16.8 MHz), is the first to support propagation and remains the most stable across the frequency range. As the frequency exceeds 30 MHz, higher-order modes such as TM₁₁ and others are activated, supporting richer but more complex signal paths.

Interpretation: The figure reinforces the waveguide nature of metro tunnels. It visually justifies why UWB systems operating in the 100–400 MHz range or above (GHz range) can exploit multiple modes for broader coverage but must also manage increased modal dispersion and interference. So, all figures shortly expressed the following: Fig. 1: Schematic of the tunnel and antenna setup, Fig. 2: Measured S12 response at different frequencies, Fig. 3: Attenuation vs. frequency at 1 m, Fig. 4: Attenuation coefficient vs. frequency, Fig. 5: Modal propagation zones.

**Table 1:** Summary of S₁₂ transmission responses at various frequencies.

Frequency (GHz)	Initial S₁₂ level (dB)	Final S₁₂ level (dB)	Propagation distance (m)	Observed pattern	Comments
1.0	− 30	− 88	6.5	Gradual decay with noise	High attenuation, possibly below cutoff for dominant modes
2.0	− 35	− 72	8.0	Smooth decay	Moderate attenuation, near cutoff; more stable than 1 GHz
3.0	− 32	− 81	6.5	Ripple/multipath pattern	Moderate loss, signs of constructive/destructive interference
4.0	− 33	− 73	8.0	Smooth and consistent	Best performance; above cutoff; ideal for tunnel systems

**Table 2:** Frequency-dependent attenuation behavior at 1 m distance.

Frequency (GHz)	Attenuation (dB/m)	Polarization	Trend	Interpretation
1.0	0.05	Horizontal	Increasing with frequency	Under-mode behavior, increasing confinement
2.0	0.098	Horizontal	Peak observed	Maximum loss point likely due to resonance
3.0	0.065	Horizontal	Decreasing after peak	Better efficiency in modal propagation
4.0	0.049	Horizontal	Continued decrease	Enhanced waveguide effect; minimal loss

**Table 3:** Comparing UWB and slot antennas in terms of attenuation and mode excitation.

Antenna type	Avg. attenuation (dB/m) at 3 GHz	Dominant mode	Bandwidth (GHz)
UWB	0.065	TE₁₀, TM₁₁	3.1–10.6
Slot	0.071	TE₁₀	2.5–4.5

### Discussion of Tables 1 and 2: EM wave propagation characteristics in Cairo metro tunnel

The data summarized in Tables 1 and 2 provides a detailed view of how electromagnetic (EM) waves behave in a closed rectangular metro tunnel environment when excited by Ultra-Wideband (UWB) antennas at different frequencies.

### Table 1: Frequency-based S₁₂ response behavior

The transmission coefficient S12, which represents the power transferred from the transmitting antenna to the receiving antenna, is shown to vary significantly with frequency and propagation distance.

- At **1 GHz**, the EM wave experiences **the highest attenuation** over a relatively short distance (6.5 m). The initial signal strength starts at − 30 dB but decays rapidly to − 88 dB, indicating that this frequency is close to or **below the cutoff frequency** for the dominant mode in this waveguide-like tunnel. The noise and irregularities in the plot at this frequency suggest potential **mode mismatches** and ineffective confinement due to suboptimal waveguide conditions.
- At **2 GHz**, signal propagation improves, with smoother attenuation across 8 m. The drop from − 35 to −72 dB demonstrates more **efficient wave confinement** and better modal propagation—likely near the tunnel’s TE₁₀ or TM₁₁ mode thresholds.
- The **3 GHz response** shows distinct **periodic amplitude variations due to multipath interference**, pointing to **multipath propagation effects** caused by wall reflections and modal interference. The signal decay is moderate, but interference causes variation in the received signal strength, particularly beyond 4 m.
- At **4 GHz**, the response exhibits the **most consistent and stable behavior**, with a linear and predictable decay from − 33 to − 73 dB over 8 m. This frequency is well above the cutoff for multiple modes in the tunnel, enabling robust and low-loss propagation, and affirming that **higher UWB frequencies are optimal for tunnel communication systems**.

### Table 2: Attenuation trends and polarization effects

Attenuation per unit distance was computed at 1 m for different frequencies under **horizontal polarization**, the dominant mode for rectangular tunnels with side-mounted slot or UWB antennas.

- **At 1 GHz**, the attenuation is 0.05 dB/m, which increases with frequency, peaking at **0.098 dB/m at 2 GHz**. This behavior reflects the **resonant absorption and maximum loss region**, where EM waves interact most inefficiently with the tunnel walls and internal structure.
- Beyond 2 GHz, attenuation **gradually decreases**, with values of 0.065 dB/m at 3 GHz and 0.049 dB/m at 4 GHz. This confirms the tunnel’s **waveguide nature**, where higher frequencies above the cutoff allow more stable mode propagation with less power loss.
- The behavior under horizontal polarization, which aligns with the geometry of TE₁₀ modes in rectangular waveguides, confirms that this configuration yields **optimal performance** in such environments. The use of horizontally polarized slot or UWB antennas is therefore validated.

### Table 3: Comparing UWB and slot antennas in terms of attenuation and mode excitation

The comparative analysis in Table 3 reveals distinct performance characteristics for UWB and slot antennas in the tunnel environment. The UWB antenna exhibits a marginally lower average attenuation (0.065 dB/m) compared to the slot antenna (0.071 dB/m) at 3 GHz. Furthermore, the UWB antenna’s ultra-wide bandwidth (3.1–10.6 GHz) allows it to excite a more diverse set of propagation modes, including both the dominant TE₁₀ and the TM₁₁ modes, which can enhance signal robustness through modal diversity. In contrast, the slot antenna, with its narrower operational bandwidth (2.5–4.5 GHz), primarily excites the fundamental TE₁₀ mode, offering a simpler but potentially less versatile propagation profile. This trade-off between lower attenuation/multi-mode capability and design simplicity is a key consideration for system designers.

The measured data supports several theoretical expectations:

1. Cutoff frequency threshold: Between 1 and 2 GHz lies a critical range where attenuation transitions from severe to manageable, consistent with modal theory for rectangular waveguides.
2. Waveguide efficiency: Frequencies above 2.5 GHz show lower attenuation and more stable responses, making them ideal for wireless systems deployed in tunnels.
3. Polarization alignment: Horizontal polarization is effective, likely due to better coupling with dominant modes and reduced wall interaction losses.
4. Multipath management: The ripple pattern at 3 GHz suggests the need for channel equalization or diversity schemes in practical systems.

## Conclusion

This study highlights the complexity of EM wave propagation in underground rectangular tunnels, particularly under UWB and slot antenna excitation. Our analysis confirms the tunnel’s behavior as a multimode waveguide and underlines the importance of selecting optimal frequencies and antenna configurations to minimize attenuation. These insights support better communication system design for underground transportation infrastructures.

## Data Availability

Data will be available on request by contacting the author M. Ibrahim via mohamed-ibrahim@du.edu.eg.
